# Empowering Caregivers: Innovative Tools for Supporting Families of People with Dementia in Community and LTC Settings

**DOI:** 10.1002/alz70858_098369

**Published:** 2025-12-25

**Authors:** Adriana Shnall

**Affiliations:** ^1^ Baycrest Centre for Geriatric Care, Toronto, ON, Canada

## Abstract

Caring for someone with dementia often leads to burnout, chronic illness, and social isolation, with caregivers facing a sixfold higher risk of developing dementia themselves. Despite these challenges, fragmented care systems and complex needs leave many resources inaccessible or iinsufficient.; To address this, Baycrest developed the Canadian Caregiver Assessment and Resource Tool (C‐CART™) and the Canadian Caregiver Adjustment Needs Tool (C‐CAN Tool™). These evidence‐based tools address two key issues: navigating care systems and supporting families during transitions to long‐term care.

C‐CART™ is a free online tool available 24/7 to help caregivers access the resources, services, education and guidance they need wherever they are located, with ease. C‐CART™ addresses the escalating challenges faced by the growing number of caregivers of all ages such as isolation, emotional burnout and financial stress, that are not currently addressed by the Canadian healthcare system. C‐CART™ uses AI‐driven technology to provide personalized recommendations. It promotes equitable access, particularly for underserved populations like rural caregivers, reducing barriers and fostering resilience.

The C‐CAN Tool™ assesses family caregivers of individuals with dementia newly admitted to residential care. This self‐administered online tool, completed shortly after admission, provides the clinical team with early insights into caregivers’ needs. Built with validated expert feedback and open‐access resources, the tool is both flexible and accessible, empowering caregivers to actively participate in care planning and collaboration with healthcare providers. Its implementation has shown potential to improve caregiver satisfaction, reduce stress, and enhance care outcomes.

These tools inform future research, including the impact of personalized interventions on caregiver burden and care quality. They also provide a foundation for policy development, emphasizing caregiver assessments in care planning across long‐term and acute care settings. By addressing caregiver and systemic needs, these tools also lay the groundwork for scalable solutions that enhance dementia care.

This presentation will identify the psychosocial needs of dementia caregivers, as well as describe the development, dissemination and practical application of innovative solutions for caregiver support such as C‐CART™ and C‐CAN Tool™ to advance dementia care. Although these are Canadian‐based tools, we will discuss how they can be adapted to an international context.

